# The synthetic synergistic cinnamon oil CIN-102 is active against *Madurella mycetomatis*, the most common causative agent of mycetoma

**DOI:** 10.1371/journal.pntd.0009488

**Published:** 2021-06-09

**Authors:** Mickey Konings, Kimberly Eadie, Wilson Lim, Ahmed H. Fahal, Johan Mouton, Nicolas Tesse, Wendy W. J. van de Sande

**Affiliations:** 1 Erasmus MC, University Medical Center Rotterdam, Department of Medical Microbiology and Infectious Diseases, Rotterdam, The Netherlands; 2 Mycetoma Research Center, University of Khartoum, Khartoum, Sudan; 3 Septeos, Research and experimental development on biotechnology, Paris, France; Universidade Federal do Para, BRAZIL

## Abstract

Mycetoma is a devastating neglected tropical infection of the subcutaneous tissue and most commonly caused by the fungus *Madurella mycetomatis*. Treatment of mycetoma consists of a combination of a long term antifungal treatment with itraconazole and surgery. However, treatment is associated with low success rates. Therefore, there is a need to identify novel treatments for mycetoma. CIN-102 is a synthetic partial copy of cinnamon oils with activity against many pathogenic bacteria and fungi. In this study we determined the *in vitro* activity of CIN-102 against 21 *M*. *mycetomatis* isolates and its *in vivo* efficacy in a *M*. *mycetomatis* infected *Galleria mellonella* larval model. *In vitro*, CIN-102 was active against *M*. *mycetomatis* with MICs ranging from 32 μg/mL to 512 μg/mL. 128 μg/mL was needed to inhibit the growth in 50% of tested isolates. *In vivo*, concentrations below the MIC of 40 mg/kg and 80 mg/kg CIN-102 prolonged larval survival, but higher concentrations of CIN-102 did not.

## Author summary

Mycetoma is a tropical infection causing large tumorous lesions on mainly the foot. This infection is most commonly caused by the fungus *Madurella mycetomatis*. Mycetoma is treated with antifungal agents and surgery but with low success rates. In our search for novel treatments for mycetoma we determined if CIN-102, a synthetic oil that resembles cinnamon oil, would be therapeutic in mycetoma. We therefore determined the activity of this oil against the fungus *in vitro*. We found that CIN-102 could inhibit *M*. *mycetomatis* growth *in vitro*. To determine if CIN-102 was also active against the mycetoma grain *in vivo*, we determined the efficacy in our *Galleria mellonella* grain model. Interestingly, CIN-102 was found to be effective in the larvae at a low concentrations.

## Introduction

Mycetoma is a chronic, granulomatous infection of the subcutaneous tissue which eventually leads to destruction of deep tissue and bone [1]. The infection is prevalent in tropical and sub-tropical regions and can be caused by either bacteria (actinomycetoma) or fungi (eumycetoma). The fungus *Madurella mycetomatis* is the most common causative agent world-wide [2]. For actinomycetoma success rates up to 90% are achieved when treated with antibiotics [1,3]. For eumycetoma a combination of surgery and prolonged antifungal medication is required, with itraconazole as the drug of choice. However, treatment of eumycetoma results in recurrence rates of 25–50%, often with greater complications, and the need for amputation in up to a quarter of patients [4,5]. This, in its turn, leads to social stigma, loss of employment and a dependency on family members. In order to improve treatment, there is a need to identify novel compounds with activity against fungal mycetoma causative agents.

In the search for new antifungal agents, natural products are seen as a rich source of possible inhibitory compounds [6]. Essential oils obtained from plants have been traditionally used in many cultures, and it has been shown that these oils contain antifungal activity [7,8]. Previous studies have already demonstrated the ability of different plant extracts and essential oils to inhibit the growth of *M*. *mycetomatis* [9,10]. Essential oils are a mixture of multiple, potentially synergistic, compounds which derive from plants as secondary metabolites. The chemical composition of these oils depends on multiple factors, such as geographical location, the environment, the maturity of the plant and the extraction method, which all subsequently effect the biological activity [11,12]. These uncertainties in the standard of quality all together limits their use clinically. Even though essential oils still have some drawbacks in clinical use, the chemical properties of most essential oils are in line with Lipinski’s rule of five on important physicochemical parameters of druggability [13,14]. Based on these oils, a more reproducible, chemically well-defined synergistic blend called CIN-102 has been developed by Septeos. CIN-102 has cinnamaldehyde as its major component (86,7% w/w) and resembles the composition of two cinnamon oils of which the genotoxins have been removed (Table 1) [15]. Henceforth, our aim is to determine if CIN-102 is able to inhibit the growth of the fungus *M*. *mycetomatis in vitro* and determine its *in vivo* efficacy within the established *Galleria mellonella* grain model [16].

**Table 1:** Chemical composition of CIN-102. A well-defined synergistic blend with antibacterial and antifungal activity.

Component	% (w/w)
Trans-cinnamaldehyde	86.7
Trans-2-methoxycinnamaldehyde	5.35
Cinnamyl acetate	2.5
Linalool	2.4
β-Caryophyllene	1.7
Cineol	1.0
Benzyl benzoate	0.35

## Methods

### Cin-102

CIN-102 is a chemical blend composed of 86.7% trans-cinnamaldehyde, 5.35% Trans-2-methoxycinnamaldehyde, 2.5% cinnamyl acetate, 2.4% linalool, 1.7% β-caryophyllene, 1.0% cineol and 0.35% benzyl benzoate (Table 1). The CIN-102 (LOT 17-110F10) used in this study was provided by Septeos (Paris, France).

### Fungal isolates

To determine if CIN-102 was able to inhibit the fungal growth of *M*. *mycetomatis*, 21 clinical isolates of *M*. *mycetomatis*, including the genome isolate MM55, were tested. These isolates originated from Sudan (n = 13), India (n = 2), Algeria (n = 1), Mali (n = 1), Somalia (n = 1) and Chad (n = 1). The origin of two isolates are unknown. All isolates were previously identified on the basis of morphology, PCR and sequencing of the internal transcribed spacer (ITS) [17,18].

### Fungal preparation and *in vitro* susceptibility testing

The antifungal activity of CIN-102 against *M*. *mycetomatis* was determined with the previously described 2,3-bis (2-methoxy-4-nitro-5-sulfophenyl)-5-[(phenylamino) carbonyl]-2H-tetrazolium hydroxide (XTT) assay [19]. In short, isolates were cultured for three weeks on Sabouraud Dextrose Agar (SDA) at 37°C after which the mycelium was harvested and sonicated for 5 seconds at 28 micron (Soniprep 150, Beun de Ronde, the Netherlands) and inoculated in RPMI 1640 medium containing 0.35 gram/Liter L-glutamine and 1.98 mM 4-morpholinepropanesulfonic acid (MOPS). The isolates were further incubated for 7 days at 37°C. The mycelium was harvested by centrifugation, washed and sonicated using the same settings. A fungal inoculum of 70% ±2% transmission (Novaspec II spectrophotometer) was prepared in RPMI 1640 medium containing 0.35 gram/Liter L-glutamine and 1.98 mM MOPS. In a 96-wells microtiter plate containing the antifungal agents in DMSO, 100 μL of the fungal suspension was added. Drug concentrations used in this assay ranged from 32 μg/mL to 16384 μg/mL for CIN-102 (Septeos, France) and from 0.008 μg/mL to 4 μg/mL for itraconazole (Janssen & Janssen, Belgium). As a growth control the fungal suspension with the solvent (1% DMSO) was used. As a negative control the culture medium with solvent but without fungal suspension was used. The plates were sealed with tape and incubated for 7 days at 37°C. After incubation, 100 μl of XTT work solution (5 mL XTT (Sigma) 1mg/mL in NaCl, 0,6 mL menadione (Sigma) 1 mM and 4,4 mL NaCl) was added to each well, followed by incubation at 37°C for 2 hours and room temperature for 3 hours. The extinction of the supernatant was measured at 450 nm in a microplate reader (Epoch2, Biotek, United States). The reduction of the absorbance was calculated and the MIC endpoints for each antifungal agent were defined as the first concentration at which spectrophotometrically at least an 80% reduction was measured. The minimal fungicidal concentration was determined by culturing the hyphal fragments of all wells where no visible growth was observed after 7 days of incubation at 37°C. These hyphal fragments were cultured on SDA at 37°C. The MFC was considered the lowest concentration of CIN-102 were no growth was observed after 2 weeks of incubation.

### *In vivo* toxicity and efficacy testing in *Galleria mellonella* larvae

In order to determine the toxicity and efficacy of CIN-102, 45 larvae/concentration were injected with CIN-102 via the left last proleg and monitored for 10 days. The tested CIN-102 concentrations were 800 mg/kg, 400 mg/kg, 80 mg/kg and 40 mg/kg solved in 10% tween-80 in PBS. To determine the efficacy of CIN-102, 15 larvae/concentration were infected with a hyphal suspension of the genome isolate *M*. *mycetomatis* MM55. To obtain a hyphal suspension, MM55 was cultured on SDA at 37°C for three weeks. The mycelium was harvested, sonicated for 30 seconds at 28 micron (Soniprep 150, Beun de Ronde, the Netherlands) and incubated in RPMI 1640 medium containing 0.35 g/L L-glutamine, 1.98 mM 4-morpholinepropanesulfonic acid (MOPS) and 100 mg/L chloramphenicol at 37°C for two weeks. To obtain the fungal mycelia, the culture was filtered through a 22 μm filter (Whatman), the biomass was scraped from the filter, weighed, re-suspended in PBS and sonicated for 2 minutes at 28 microns. Followed by centrifugation at 3400 rpm for 5 minutes, the biomass was adjusted with PBS to a concentration of 1 g/10 mL. Larvae were injected with 40 μl of inoculum via the last left pro-leg, resulting in a final concentration of 4 mg wet weight of fungi per larvae. Larvae were divided into groups of 15 and each group was treated on three consecutive days at 4, 28 and 52 hours post-infection. Administered dosages consisted of either 40 mg/kg, 80 mg/kg, 400 mg/kg or 800 mg/kg CIN-102 solved in 10% tween-80 in PBS. Additionally, 5.71mg/kg itraconazole (which results to a human equivalent pharmacokinetic dosages) and solvent only (10% tween-80 in PBS) control groups were included [3]. Larvae survival was monitored for 10 days. Three biological replications were performed.

### Histological examination of *M*. *mycetomatis* infected larvae and assessment of the fungal burden

To determine the fungal burden within the larvae, larvae were infected and treated as described above. At 24 and 72 hours post-infection, five larvae from each group were sacrificed for histological examination and assessment of fungal burden. The larvae were fixed in 10% buffered formalin for 24 hours, dissected longitudinally into two halves with a scalpel and fixed for another 48 hours before further routine histological processing [20]. The two halves were stained with hematoxylin and eosin (HE) and Grocott methanamine silver for further histological examination. Assessment of the fungal burden was performed by manually counting of the grains by three independent scientists, under a light microscope mounted with a Canon EOS70D camera (Canon Inc). Visualization of the grains was done on the computer screen using EOS Utility software (Canon Inc.). Under 40x magnification with a light microscope, the grains were categorized into large (>0.02mm^2^), medium (0.01–0.019 mm^2^) and small (0.005–0.009 mm^2^) sizes as described by Lim et al. [21]. The sum of all large, medium and small grains represents the total amount of grains observed within the larvae. In order to determine the total size of the grains, the sum of the all grains were multiplied by the minimum size of their respected categories [21].

### Statistical analysis

To compare the survival lines and determine if there was a statistical difference between the different treatment groups, the Log-rank test was performed with GraphPad Prism 8 (version 8.2.0, GraphPad Inc.). A Mann-Whitney test was performed to determine if the number of grains or the size of the grains within the larvae differed between larvae treated with CIN-102, ITZ or solvent. A p-value smaller than 0.05 was deemed significant.

## Results

### *In vitro M*. *mycetomatis* is inhibited by CIN-102

**Fig 1:**
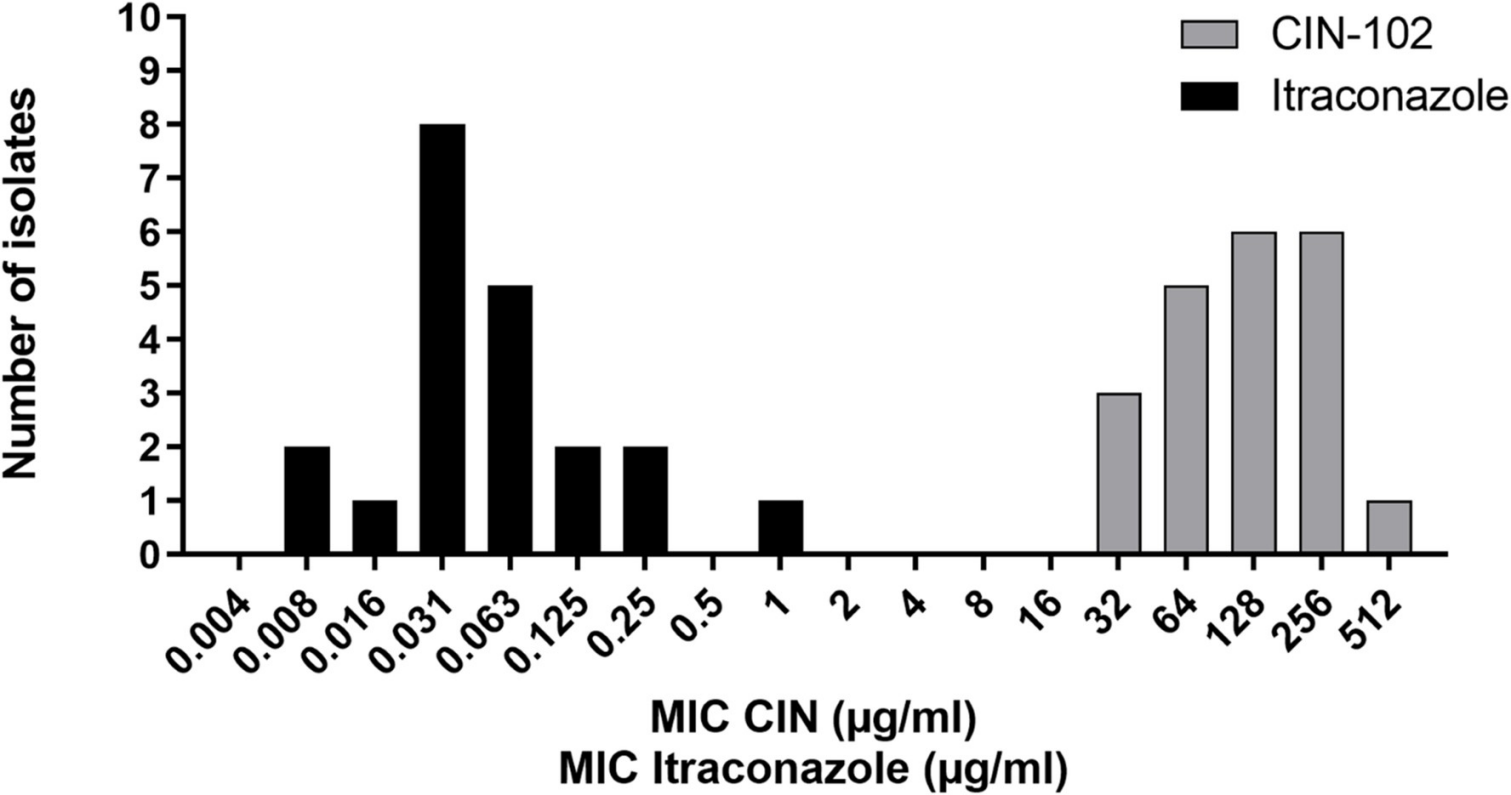
Antifungal susceptibility of 21 *M*. *mycetomatis* isolates to CIN-102 and itraconazole expressed as MICs, determined by the XTT assay.

As shown in Fig 1, MIC values for itraconazole ranged from <0.008 μg/mL to 1 μg/mL and those for CIN-102 ranged from <32 μg/mL to 512 μg/mL, this corresponds to <28 μg/mL- 444 μg/mL cinnamaldehyde (Table 2). The MIC for genome isolate MM55 was 256 μg/ml, this corresponds to 222 μg/mL cinnamaldehyde. The MIC50 and MIC90 of itraconazole were 0.03 μg/mL and 0.25 μg/mL respectively. To inhibit the growth of all tested isolates, 1 μg/mL itraconazole was required For CIN-102, the MIC50 and MIC90 were 128 μg/mL CIN-102 (111 μg/mL cinnamaldehyde) and 256 μg/mL CIN-102 (222 μg/ml cinnamaldehyde). 512 μg/mL CIN-102 was required to inhibit the growth of all tested isolates. For CIN-102, the observed minimal fungicidal concentrations were similar to the respective MIC values.

**Table 2:** *In vitro* susceptibility of all included *M*. *mycetomatis* isolates to CIN-102 and itraconazole expressed in μg/mL. The country of isolation and accession number for each isolate is s The corresponding concentrations of cinnamaldehyde have been calculated and shown in μg/mL. The MIC ranges, MIC50 and MIC90 to *M*. *mycetomatis* are shown in μg/mL.

Isolate	Country of isolation	Accession number ITS region	CIN-102 (μg/mL)	Cinnamaldehyde (μg/mL)	Itraconazole (μg/mL)
MM13	Sudan	JX280866.1	256	222	0.06
MM14	Sudan	MW513510	128	111	0.03
MM25	Sudan	MW494400	128	111	0.03
MM26	Sudan	MW538038	128	111	0.25
MM30	Sudan	MW520456	64	56	0.06
MM35	Sudan	MW513693	64	56	0.03
MM36	Sudan	MW520454	64	56	0.03
MM41	Sudan	MW520455	256	222	0.03
MM44	Sudan	MW520452	32	28	0.03
MM52	Sudan	JN573179.1	64	56	<0.008
MM54	Sudan	JN573180.1	32	28	0.016
MM55	Sudan	JN573181.1	256	222	0.06
MM56	Sudan	JN573182.1	32	28	<0.008
AL1	Algeria	MW541888	64	56	0.03
SO1	Somalia	MW493233	512	444	0.06
P1	Mali	MW520453	128	111	0.06
I1	India	JX280864.1	256	222	1
I11	India	MW541890	128	111	0.125
CBS116298	Chad	MW542679	256	222	0.125
CBS247.48	unknown	JX280745.1	256	222	0.25
t606931	Unknown	MW541889	128	111	0.03
MIC range			**32–512**	**28–444**	**<0.008–1**
MIC_50_			**128**	**111**	**0.03**
MIC_90_			**256**	**222**	**0.25**

### Administering sub-MIC concentrations CIN-102 significantly prolongs larval survival

**Fig 2:**
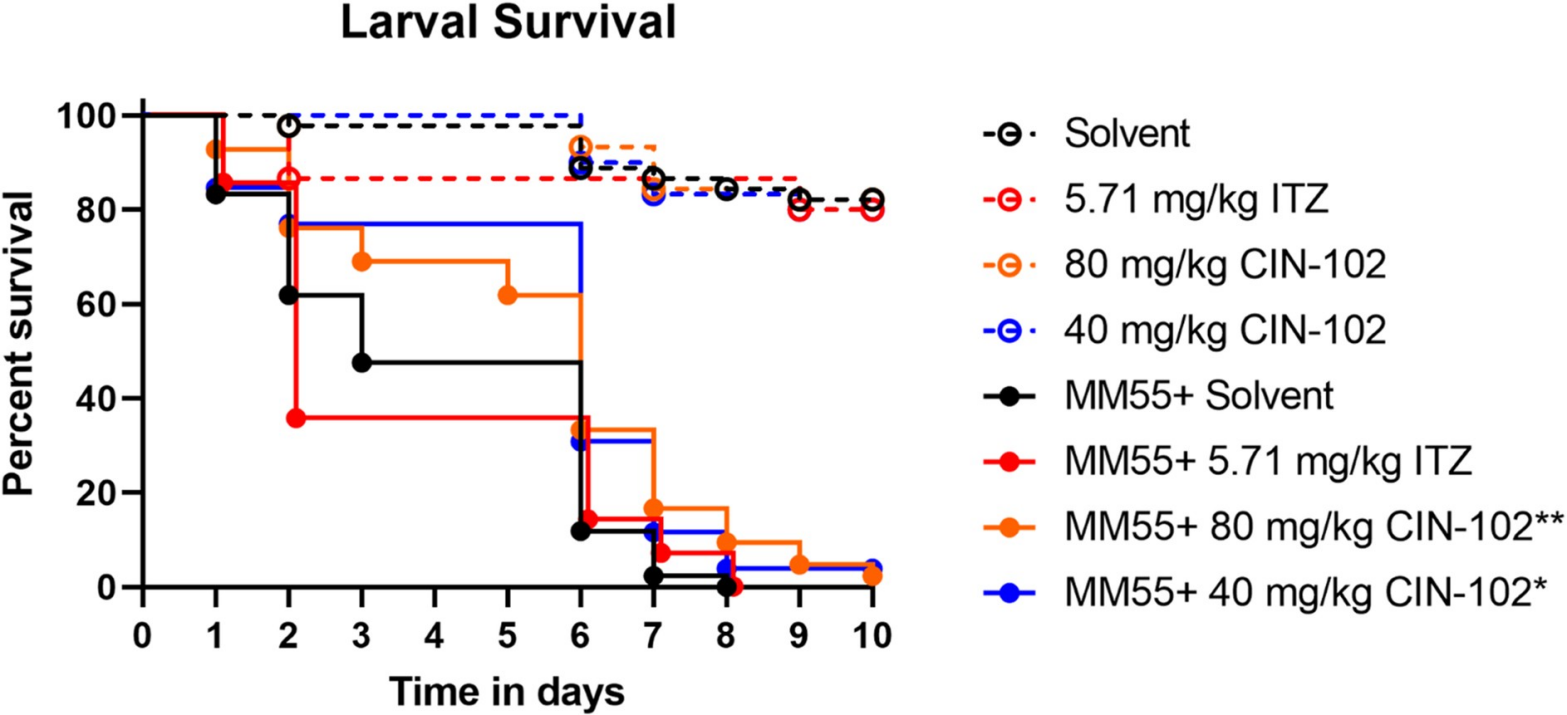
*In vivo* efficacy and toxicity of different dosages CIN-102 and the used treatment dosage itraconazole within *G*. *mellonella* larvae, compared to the solvent (PBS 10% tween80). Dotted lines indicate uninfected larvae as toxicity controls, which were treated at the same time points as the infected groups. It is observed that none of the shown dosages effect the survival of uninfected larvae. The full lines indicate infected larvae treated with the corresponding dosages 4-, 28- and 52-hours post-infection. Larvae treated with solvent and itraconazole survive up until day 8, while larvae treated with 40 mg/kg and 80 mg/kg survive up until day 10 and significantly differ from the PBS control (*p = 0.0133**p = 0.0091).

As can be seen in S1 Fig, after multiple dosing the highest concentration of 800 mg/kg CIN-102 (694 mg/kg cinnamaldehyde) appeared to be toxic to the larvae and resulted in a decreased survival in uninfected as well as infected larvae (Log-Rank, p = 0.0003 and p = 0.03 respectively). No signs of toxicity were noted with 40, 80 and 400 mg/kg (S1 Fig). CIN-102 concentrations at 40 mg/kg (35 mg/kg cinnamaldehyde) and 80 mg/kg (70 mg/kg cinnamaldehyde) resulted in a significantly enhanced larvae survival (Fig 2)(Log-Rank, p = 0.0133 and p = 0.0091, respectively). 5.71 mg/kg Itraconazole did not enhance larvae survival (Log-Rank, p = 0.77), which was in agreement with our earlier findings [22,23].

### CIN-102 does not significantly reduce the number or the size of the grains

**Fig 3:**
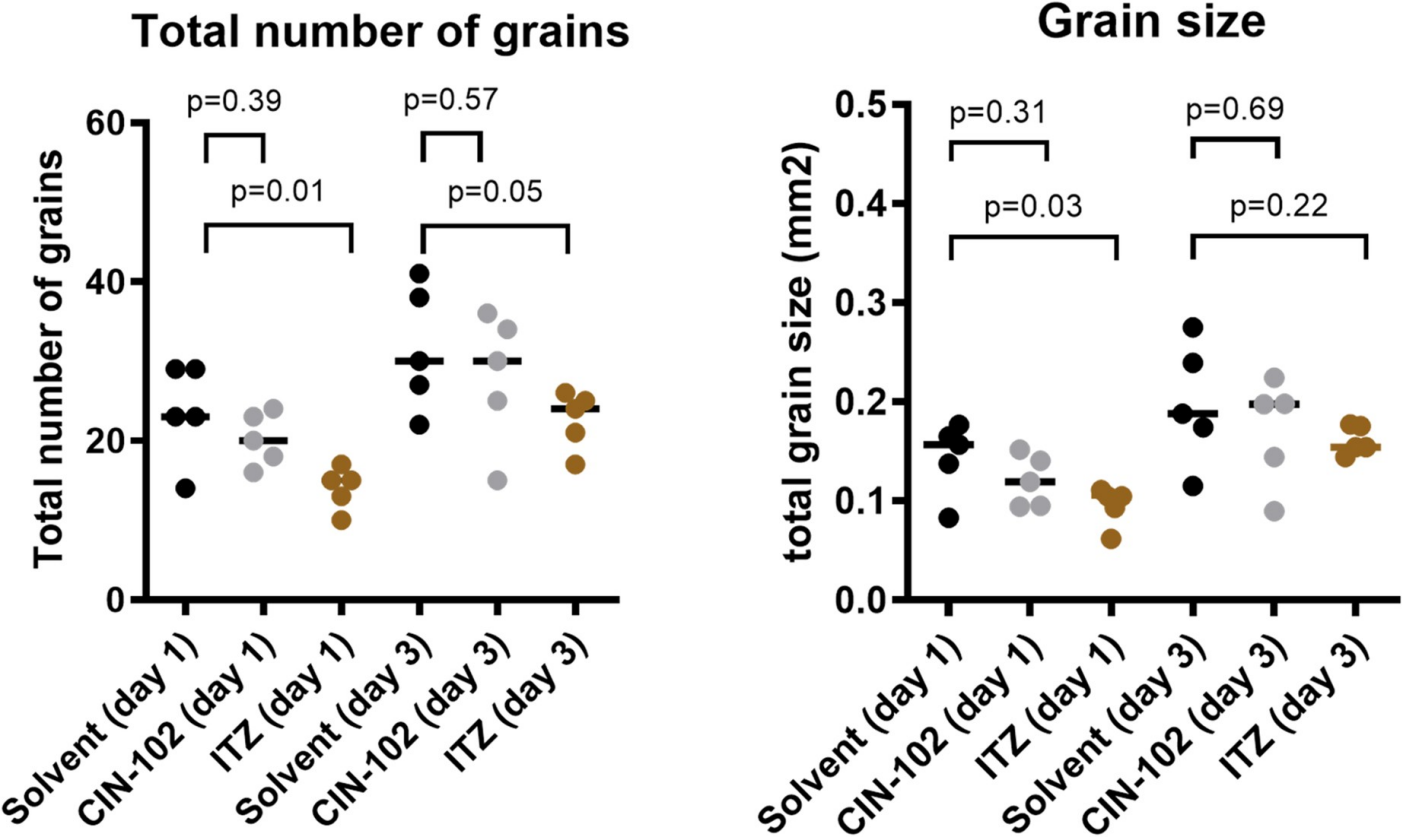
The total number and size of grains in *M*. *mycetomatis* infected *G*. *mellonella* larvae treated with solvent (black dots), 80 mg/kg CIN-102 (grey dots), or 5.71mg/kg itraconazole (brown dots). Total number of grains counted in CIN-102 treated larvae do not differ at both one- and three-days post-infection (Man-Whitney, p = 0.39 and p = 0.57 respectively). The same is found for total grain size, for which no difference is found between CIN-102 treated larvae and the control, both one- and three-days post-infection (Man-Whitney, p = 0.31 and p = 0.69 respectively). Total number of grains counted in itraconazole treated larvae differed both one- and three-days post-infection (Man-Whitney, p = 0.01 and p = 0.05 respectively). Similarly, the total grain size differed between itraconazole and the control in both one- and three-days post-infection (Man-Whitney, p = 0.03 and p = 0.22 respectively).

To assess the fungal burden within the larvae, the number and the size of the grains were determined by histological examination 24 and 72 hours after fungal infection. As shown in Fig 3, no difference in either the total number or in the total size of the grains was noted 24 and 72 hours after infection between larvae treated with 80 mg/kg CIN-102 and the control (Mann-Whitney, total number of grains p = 0.39 and p = 0.57 and total grain size p = 0.31 and p = 0.69 after 1 and 3 days respectively).For itraconazole, a reduction in the total number and the total size was noted (Mann-Whitney, total number of grains p = 0.01 and p = 0.05 and total grain size p = 0.03 and p = 0.22 after 1 and 3 days respectively), which was in agreement with our earlier findings [21].

**Fig 4:**
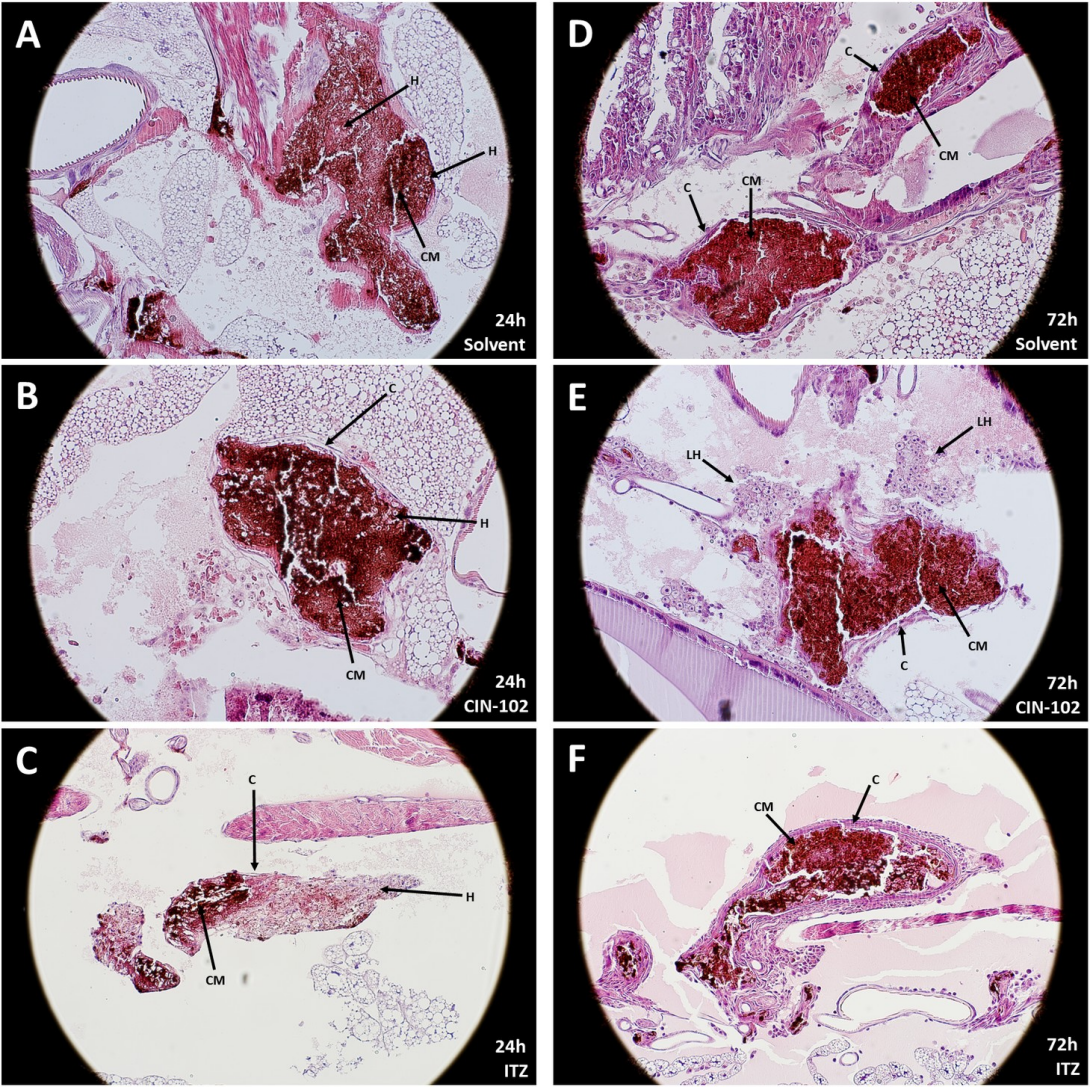
*M*. *mycetomatis* grains in *Galleria mellonella* larvae, 200x magnification. **A:** *M*. *mycetomatis* grain 24h after infection and treated with solvent. Cement material (CM) is present. No clear capsule is formed surrounding this grain. Some individual hemocytes (H) are present in the cement material. **B:** *M*. *mycetomatis* grain 24h after infection and treated with 80mg/kg CIN-102. Cement material is present. A capsule (C) is surrounding the grain, individual hemocytes are found in the grain. **C:** *M*. *mycetomatis* grain 24h after infection and treated with 5.71 mg/kg itraconazole (ITZ). Partial formation of cement material and encapsulation of the grain are observed. Individual hemocytes (H) are present in the cement material. **D:** *M*. *mycetomatis* grain 72h after infection and treated with solvent. Dense cement material seen, no individual hemocytes inside the grain. A capsule is surrounding the grain. **E:** *M*. *mycetomatis* grain 72h after infection and treated with 80mg/kg CIN-102. Cement material is less dense compared to the control (panel C). Capsule is surrounding the grain. Some large hemocytes (LH) are seen surrounding the grain and is not seen in solvent or itraconazole treated larvae. **F:** *M*. *mycetomatis* grain 72h after infection and treated with 5.71 mg/kg itraconazole (ITZ). Both cement material (CM) and encapsulation (C) of the grain are observed.

Although treatment with CIN-102 did not result in a difference in the size and number of grains, differences were observed regarding the physiology surrounding the fungal grain. After treatment with 80 mg/kg CIN-102, encapsulation occurred earlier and a decrease in the cement material was observed compared to the controls. Similar findings were observed for itraconazole. However, unique to treatment with CIN-102 was the observation of hemocytes with a larger morphology surrounding the grain. These were not present in the control group or the itraconazole treated group (Fig 4).

## Discussion

In this experimental work we explored the possibility of the synthetically well defined, synergistic blend CIN-102 as a potential novel therapeutic for mycetoma. As demonstrated in this study, CIN-102 was able to inhibit *M*. *mycetomatis* growth with MICs ranging from 32 μg/mL (28 μg/mL cinnamaldehyde) to 512 μg/mL (444 μg/mL cinnamaldehyde). A concentration of 256 μg/mL (222 μg/mL cinnamaldehyde) was needed to inhibit the growth of 90% (MIC_90_) of the isolates. This was comparable to the activities found of different essential oils generated from *Cinnamomum* spp. against other fungal species where for most of these essential oils, 40 μg/mL to 310 μg/mL was needed to inhibit the growth of *Candida* spp., *Cryptococcus neoformans*, *Trichophyton* spp., *Microsporum* spp. and *Aspergillus fumigatus* determined by broth microdilution following CLSI guidelines [24]. The main antifungal compound of CIN-102, cinnamaldehyde, has been described with comparable inhibitory activities as well. A concentration of 100 μg/mL to 500 μg/mL (determined by microdilution) and 40 μg/mL to 138 μg/mL (determined by disc diffusion and gradient plate method) was sufficient for the inhibition of *Candida* spp. and *Aspergillus* spp. growth, respectively [25–27].

CIN-102 was able to prolong larval survival in *M*. *mycetomatis* infected larvae. Within these larvae, characteristic mycetoma grains are formed. Furthermore, the antifungal efficacy studies performed in this model appeared to be predictive of the efficacy studies performed in mice [23,28]. Interestingly, prolonged larval survival was only noted when larvae were treated with relatively low concentrations of CIN-102 and not with the higher concentration of 400 mg/kg. In fact, the effective dosages of 40 mg/kg and 80 mg/kg CIN-102 were below the MIC of 256 μg/ml (corresponds to 256 mg/kg) obtained for genome isolate MM55 from our *in vitro* data. Furthermore, despite the enhanced survival, no reduction in the total number of grains or the size of the grains was noted. The observation of earlier encapsulation of the grains and enlarged hemocytes in the larvae suggested a possible influence of CIN-102 on the *G*. *mellonella* immune response. A limitation of the current study is that we did not investigate the immune reaction of the *G*. *mellonella* larvae towards CIN-102. However, some clues can be obtained from literature. Cinnamaldehyde, the major compound of CIN-102, has been described to up-regulate expression and production of anti-inflammatory cytokines IL-10 and TGF-β, while down-regulating pro-inflammatory cytokine expression and production [29]. CIN-102 component 2-methoxycinnamaldehyde has been described to inhibit the pro-inflammatory cytokine TNF-α, while anti-inflammatory properties have been described for CIN-102 components linalool, β-caryophyllene and cineol [30–33]. It was shown that upon CIN-102 treatment, decreased mRNA levels of the pro-inflammatory cytokines IL-1β and TNF-α and increased expression of IL-6 was noted in colonic tissue in mice suffering from colitis. Although it was not clear if the decrease in infection was directly linked to the antimicrobial effect of CIN-102, or as an indirect result of CIN-102 its anti-inflammatory effect [15].

The importance of the host immune system in treatment outcome was recently demonstrated by the remarkable cure of a mycetoma patient upon the addition of non-steroidal anti-inflammatory drug (NSAID) diclofenac to the treatment alongside posaconazole and 5-flucytosine [34]. It is therefore plausible that the observed enhanced survival of the *G*. *mellonella* larvae was not only due to the immediate fungicidal effect of CIN-102 to the fungus but the combination of fungicidal activity and an altered immune response of the larvae towards the mycetoma grain.

In conclusion, CIN-102 showed promising *in vitro* potency against *M*. *mycetomatis* and *in vivo* efficacy was demonstrated within *M*. *mycetomatis* infected *Galleria mellonella* larvae. This study shows the potential of chemically well-defined blends, which offer an interesting alternative to current single-drugs screening approaches. As mentioned earlier, this study was not designed to evaluate the immunological response upon treatment. Following this major limitation, more studies are required to determine the mechanism of action of CIN-102, the potential of other synergistic blends and the involvement of the immune response during fungal mycetoma infections.

## Supporting information

S1 Fig *In vivo* toxicity (panel A) and efficacy (panel B) of different dosages CIN-102 and the used treatment dosage itraconazole within *G*. *mellonella* larvae, compared to the solvent (PBS 10% tween80). In panel A, A concentration of 800 mg/kg CIN-102 is significantly different from the solvent control (***p = 0.0003), none of the other dosages effect the survival of uninfected larvae. In panel B, the dotted line indicates uninfected larvae as toxicity controls, which were treated at the same time points as the infected groups. The full lines indicate infected larvae treated with the corresponding dosages 4-, 28- and 52-hours post-infection. Larvae treated with solvent and itraconazole survive up until day 8, while larvae treated with both 40 mg/kg and 80 mg/kg survive up until day 10 and significantly differ from the PBS control (*p = 0.0133 and **p = 0.0091).
(TIF)
