# Supplementary figures and images for: The synthetic synergistic cinnamon oil CIN-102 is active against Madurella mycetomatis, the most common causative agent of mycetoma
Source: PLoS Negl Trop Dis. 2021 Jun 9;15(6):e0009488. doi: 10.1371/journal.pntd.0009488 (PMC8216527; doi:10.1371/journal.pntd.0009488)

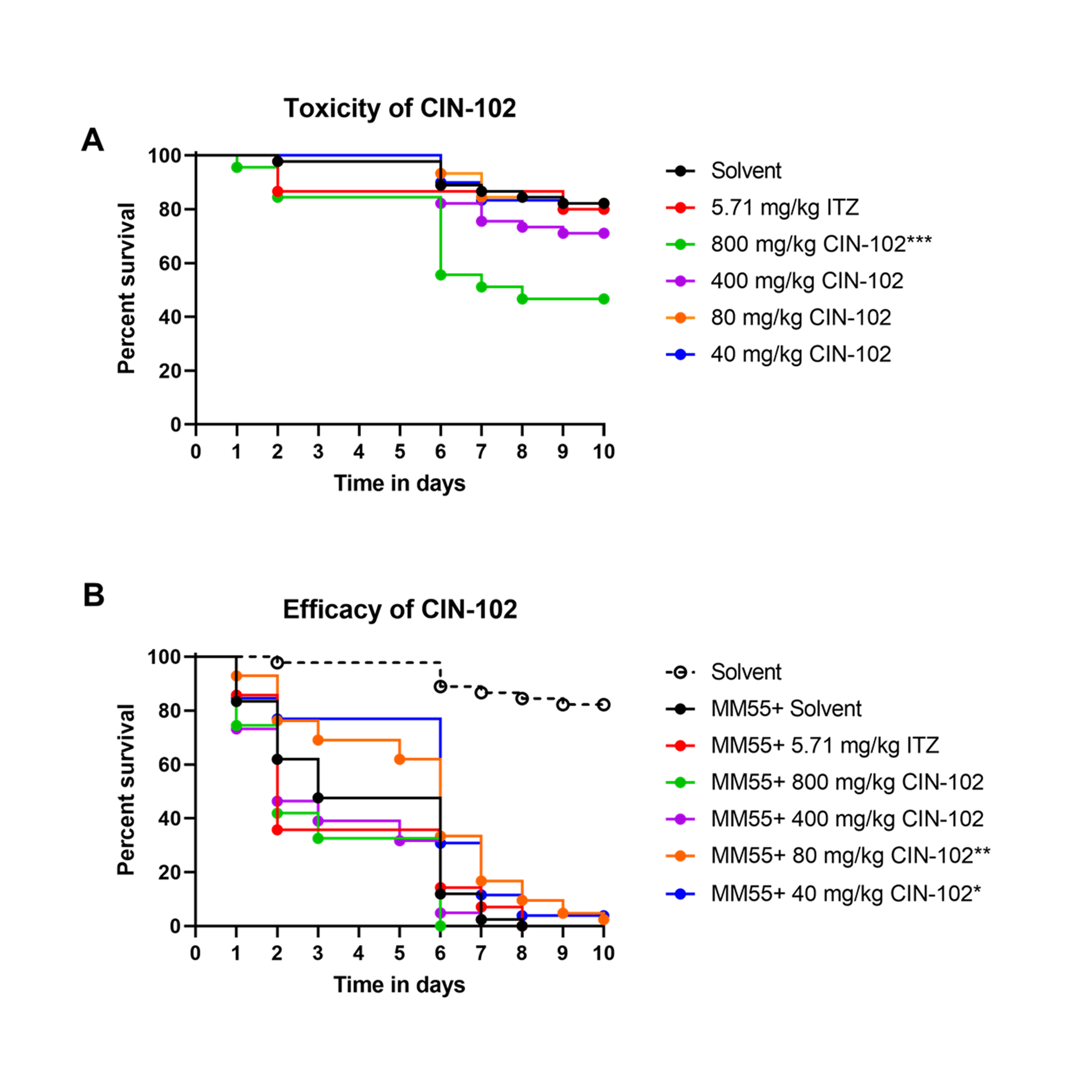

Supplement: S1 Fig — In vivo toxicity (panel A) and efficacy (panel B) of different dosages CIN-102 and the used treatment dosage itraconazole within G. mellonella larvae, compared to the solvent (PBS 10% tween80). In panel A, A concentration of 800 mg/kg CIN-102 is significantly different from the solvent control (***p = 0.0003), none of the other dosages effect the survival of uninfected larvae. In panel B, the dotted line indicates uninfected larvae as toxicity controls, which were treated at the same time points as the infected groups. The full lines indicate infected larvae treated with the corresponding dosages 4-, 28- and 52-hours post-infection. Larvae treated with solvent and itraconazole survive up until day 8, while larvae treated with both 40 mg/kg and 80 mg/kg survive up until day 10 and significantly differ from the PBS control (*p = 0.0133 and **p = 0.0091). (TIF) [file pntd.0009488.s001.tif]
